# Zebrafish *gbx1 *refines the Midbrain-Hindbrain Boundary border and mediates the Wnt8 posteriorization signal

**DOI:** 10.1186/1749-8104-4-12

**Published:** 2009-04-02

**Authors:** Muriel Rhinn, Klaus Lun, Reiner Ahrendt, Michaela Geffarth, Michael Brand

**Affiliations:** 1 Biotechnology Center, and Center for Regenerative Therapies Dresden, CRTD, Dresden University of Technology, Tatzberg 47-51, 01307 Dresden, Germany; 2 Current address: Institut de Génétique et de Biologie Moléculaire et Cellulaire, CNRS/INSERM/ULP, UMR 7104, BP 10142, CU de Strasbourg, 67404 Illkirch Cedex, Strasbourg, France; 3 Current address: Research Division Leica Microsystems Wetzlar GmbH, Ernst-Leitz Strasse 17-37, 35578 Wetzlar, Germany

## Abstract

**Background:**

Studies in mouse, *Xenopus *and chicken have shown that *Otx2 *and *Gbx2 *expression domains are fundamental for positioning the midbrain-hindbrain boundary (MHB) organizer. Of the two zebrafish *gbx *genes, *gbx1 *is a likely candidate to participate in this event because its early expression is similar to that reported for *Gbx2 *in other species. Zebrafish *gbx2*, on the other hand, acts relatively late at the MHB. To investigate the function of zebrafish *gbx1 *within the early neural plate, we used a combination of gain- and loss-of-function experiments.

**Results:**

We found that ectopic *gbx1 *expression in the anterior neural plate reduces forebrain and midbrain, represses *otx2 *expression and repositions the MHB to a more anterior position at the new *gbx1*/*otx2 *border. In the case of *gbx1 *loss-of-function, the initially robust *otx2 *domain shifts slightly posterior at a given stage (70% epiboly), as does MHB marker expression. We further found that ectopic juxtaposition of *otx2 *and *gbx1 *leads to ectopic activation of MHB markers *fgf8*, *pax2.1 *and *eng2*. This indicates that, in zebrafish, an interaction between *otx2 *and *gbx1 *determines the site of MHB development. Our work also highlights a novel requirement for *gbx1 *in hindbrain development. Using cell-tracing experiments, *gbx1 *was found to cell-autonomously transform anterior neural tissue into posterior. Previous studies have shown that *gbx1 *is a target of Wnt8 graded activity in the early neural plate. Consistent with this, we show that *gbx1 *can partially restore hindbrain patterning in cases of Wnt8 loss-of-function. We propose that in addition to its role at the MHB, *gbx1 *acts at the transcriptional level to mediate Wnt8 posteriorizing signals that pattern the developing hindbrain.

**Conclusion:**

Our results provide evidence that zebrafish *gbx1 *is involved in positioning the MHB in the early neural plate by refining the *otx2 *expression domain. In addition to its role in MHB formation, we have shown that *gbx1 *is a novel mediator of Wnt8 signaling during hindbrain patterning.

## Background

Patterning of the vertebrate neural plate depends on the formation of local organizing centers that release effector molecules to control the regionalization of adjacent tissue. The first step in establishing an organizing center is proposed to be the specification of two distinct adjacent cell populations that subsequently undergo local cell-cell interactions to influence cell fate [1]. This model applies in part to the formation of the midbrain-hindbrain boundary (MHB) organizer, a source of signals that direct regional specification of both the midbrain and anterior hindbrain (reviewed in [2,3]). In mouse, the MHB arises from subdivision of the early neural plate into two domains that express distinct transcription factors: an anterior *Otx2 *expressing domain that encompasses both forebrain and midbrain primordia [4-6], and a posterior *Gbx2 *expressing domain that encompasses presumptive anterior hindbrain [7]. Intriguingly, *gbx *function during MHB formation in zebrafish does not appear to be carried out by *gbx2*. We have previously shown that *gbx2 *is expressed too late for such a role [8], and loss-of-function assays suggest that *gbx2 *functions in MHB maintenance, rather than formation [9]. Thus, it remains unknown what gene in zebrafish acts alongside *otx2 *to subdivide the neural plate early in MHB formation. Here we investigate a potential role for zebrafish *gbx1*, the other *gbx *homolog, during early neural patterning, and find that it has an important function during MHB formation as well as a novel role in the developing hindbrain.

Proper MHB organizer formation is defined by a cascade of genetic interactions that are marked by complex temporo-spatial patterns of gene expression. At the end of gastrulation, the transcription factors *Pax2*/*5 *and *En1*/*2*, and the secreted molecules Wnt1 and Fgf8 are expressed at the *Otx2*/*Gbx2 *interface (reviewed in: [2,10,11]). Studies in mice, chicken, and zebrafish indicate that three parallel signaling pathways, involving *Pax2*/*pax2.1*, *Wnt1 *and *Fgf8*, are activated independently at this interface [12,13] (reviewed in [10,14]). These three pathways become mutually dependent within a regulatory loop that is crucial for continued MHB development and its signaling activities during somitogenesis (reviewed in [2,10]). *Otx2 *or *Gbx2 *are necessary for correct antero-posterior (A-P) positioning of *Fgf8 *and *Pax2 *expression domains in mouse, although they are not required to initiate expression [7,15-19]. In *Otx2 *or *Gbx2 *loss-of-function mutants, this loss of positional information is reflected in the formation of large overlapping *Fgf8 *and *Pax2 *domains [7,15-19].

Mutual antagonism between *Otx2 *and *Gbx2 *has been shown to determine MHB position in mouse [17,20] and *Xenopus *[21,22]. *Gbx2 *misexpression in the caudal midbrain under the control of the *Wnt1 *promoter represses *Otx2 *expression and shifts the MHB organizer rostrally [17]; conversely, *Otx2 *misexpression in the rostral hindbrain under the *En1 *promoter represses *Gbx2 *expression and shifts the MHB posteriorly [20]. In both cases, *Fgf8 *expression is localized at the new *Otx2*/*Gbx2 *interface. Studies in chicken also have shown that ectopic juxtaposition of *Gbx2 *and *Otx2 *expression domains can induce MHB marker expression [23-25]. In sum, these experimental data suggest that negative interactions between *Otx2 *and *Gbx2 *generate a sharp boundary between their two expression domains and that the region where *Otx2 *and *Gbx2 *abut might demarcate the MHB primordium (reviewed in [10,11,26-28]).

Similar to *Gbx2 *expression in mouse [7,29], zebrafish *gbx1 *is expressed in the prospective hindbrain/spinal cord, adjacent to the *otx2 *domain, from the onset of gastrulation (70% epiboly) [8]. Additionally, all known players in the maintenance and/or organizing activity of the MHB (*pax2.1*/*5*, *eng1*/*2*, *wnt1*, *fgf8*) start to be expressed on either one or both sides of the *gbx1*/*otx2 *border. In contrast to zebrafish, mouse *Gbx1 *is never expressed at the MHB [30,31] and, as of yet, no functional data concerning *Gbx1 *are available.

In the present study, we set out to ascertain the function of *gbx1 *during early development in zebrafish. We show that ectopic *gbx1 *expression represses *otx2 *and shifts the MHB anteriorly to sit at the newly created *gbx1*/*otx2 *interface. Conversely, loss of *gbx1 *function results in a slight posterior expansion of the *otx2 *domain at 70% epiboly that is accompanied by a shift in MHB markers. These results provide evidence that, in zebrafish, *gbx1 *is playing an analogous role to *Gbx2 *in terrestrial vertebrates in positioning the MHB in the early neural plate by refining the *otx2 *expression domain.

Zebrafish *gbx1 *is also known to be expressed in the hindbrain territory during gastrulation [8], suggesting that it may have an additional function in hindbrain development. In agreement with this, we find that *gbx1 *gain- and loss-of-function affects the hindbrain territory as well as the MHB. Using cell tracing experiments we show that *gbx1 *induces posterior neural cell fate via cell-autonomous transformation of anterior neural tissue. Given that *gbx1 *is a target of the posteriorizing signal Wnt8 [32], and our finding that *gbx1 *overexpression rescues hindbrain loss in cases of Wnt8 loss-of-function, we conclude that in addition to its role in MHB formation, *gbx1 *is a novel mediator of Wnt8 signaling during hindbrain patterning.

## Results

### Ectopic *gbx1 *represses *otx2 *expression and expands the hindbrain

**Figure 1:**
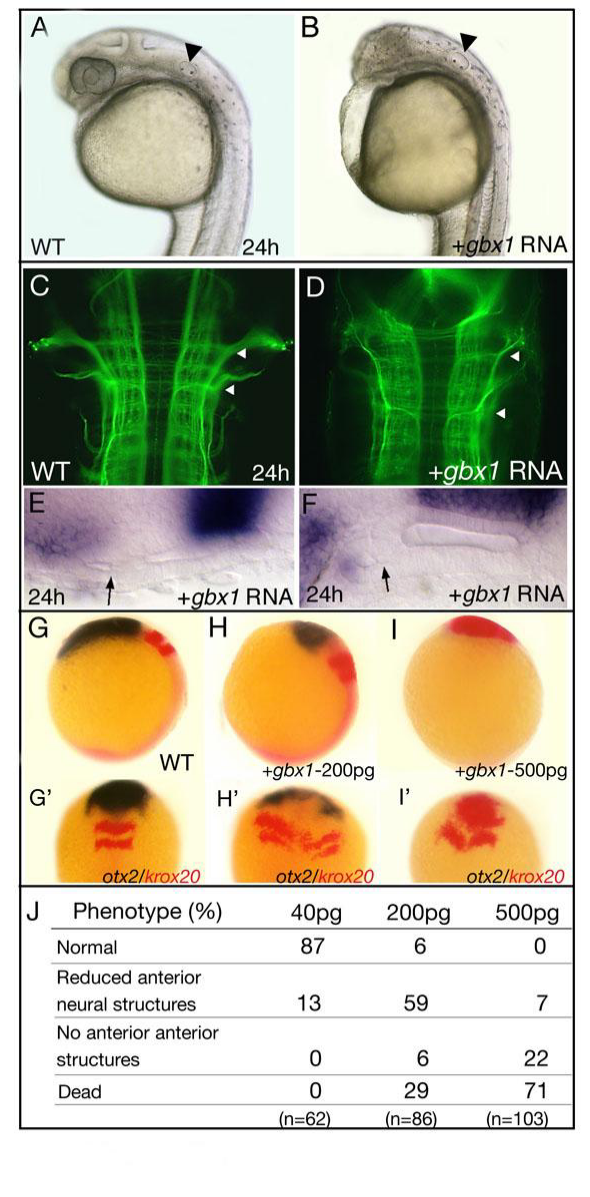
**Overexpression of *gbx1 *induces posterior cell fate**. **(A) **Wild-type (WT) embryo at 24 h and **(B) **after injection of 200 pg *gbx1 *mRNA; anterior brain structures are severely reduced. The ear is indicated by an arrowhead. **(C, D) **Staining of the forming axon tracts with an anti-acetylated tubulin antibody at 24 h; dorsal views, with anterior to the top. In the *gbx1*-injected embryo the hindbrain is severely enlarged compared to WT (arrowheads). **(E, F) **Duplications of ear structures are frequently observed (arrows). **(G-I') **Series of embryosinjected with different doses of *gbx1 *mRNA (200 and 500 pg), analyzed at the tailbud stage after *in situ *hybridization with *otx2 *(blue) and *krox20 *(red). The *otx2 *domain progressively disappears and the *krox20 *domains shift to more anterior regions. **(J) **Dose-dependent *gbx1 *overexpression phenotypes. Higher concentrations (>500 pg) did not increase the observed phenotype. (A-D, K-M) Lateral views; (E-H, K'-M') dorsal views.

To study *gbx1 *function within the zebrafish early neural plate, we first injected synthetic *gbx1 *mRNA into one-cell stage embryos. The injected embryos showed an enlarged hindbrain and a strong reduction of anterior neural structures, lacking eyes, fore- and midbrain at 24 h (Figure 1A, B; see also [9]). Staining of early axon tracts with an anti-acetylated tubulin antibody revealed that this enlarged hindbrain was specified normally (Mauthner neuron and trigeminal ganglion are present; Figure 1C, D, arrowheads). Apart from the posteriorized neural phenotype, *gbx1 *overexpressing embryos also produced additional ear structures (Figure 1E, F, arrows).

**Figure 2:**
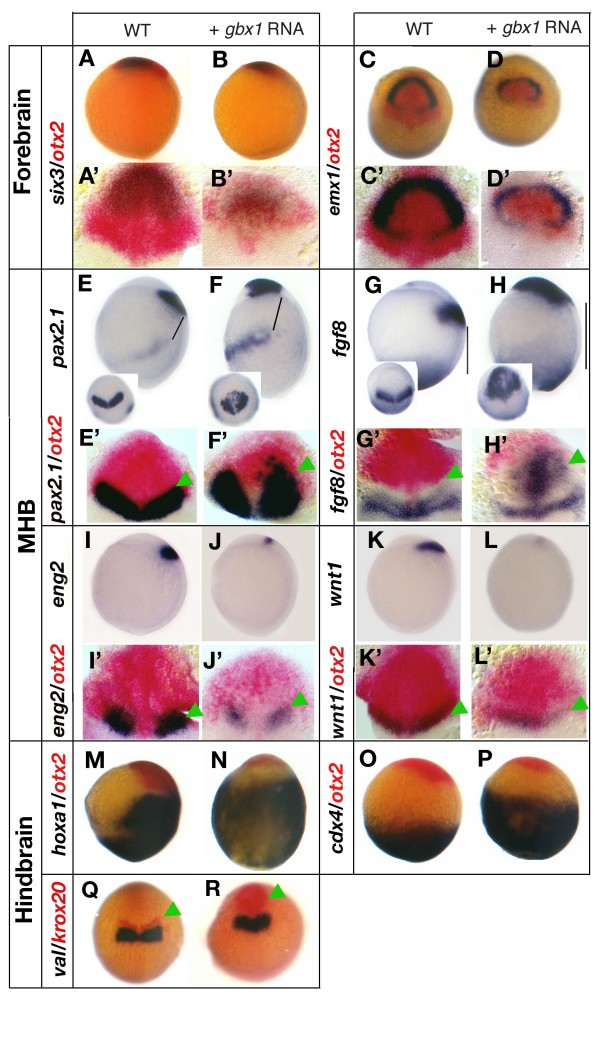
***gbx1 *overexpression affects midbrain, midbrain-hindbrain boundary (MHB) and hindbrain**. **(A, A') **Control embryo stained for *six3 *(blue) and *otx2 *(red);. **(B, B') ***gbx1*-injected embryo. The near absence of *otx2*-positive cells posteriorly to the *six3*-expressing domain indicates a loss of the midbrain territory in the *gbx1*-injected embryos. **(C, C') **Control embryo stained for *emx1 *(blue) and *otx2 *(red). **(D, D') ***gbx1*-injected embryo. The *emx1 *domain corresponding to forebrain remains robust, whereas the *otx2 *domain corresponding to midbrain is lost. **(E) **Wild-type (WT) embryo after *in situ *hybridization (ISH) with *pax2.1 *and **(E') **with *pax2.1 *and *otx2 *(red). **(F, F') ***gbx1*-injected embryos; *pax2.1 *is expressed ectopically in the anterior of the embryo. The MHB is shifted anteriorly as indicated by green arrowheads. Also, the distance between the posterior *pax2.1 *domain and the anterior domain is increased (black bars in (E, F)). **(G) **WT embryo after ISH with *fgf8 *and **(G') **with *fgf8 *and *otx2 *(red). **(H, H') ***gbx1*-injected embryos; *fgf8 *is expressed ectopically in the anterior of the embryo. The MHB is shifted anteriorly as the distance between this expression domain and the margin in increased (black bars in (G, H)). **(I) **WT embryo after ISH with *eng2 *and **(I') **with *eng2 *and *otx2 *(red). **(J) ***gbx1*-injected embryo; *eng2 *is not expressed ectopically. **(J') **Combined ISH with *otx2 *(red) shows that the remaining domain is located caudally to the reduced *otx2 *domain. **(K) **WT embryo after ISH with *wnt1 *and **(K') **with *wnt1 *and *otx2 *(red). **(L) ***gbx1*-injected embryo;*wnt1 *is not expressed ectopically. **(L') **In all *gbx1*-injected embryos the MHB is shifted anteriorly as indicated by green arrowheads. **(M) **WT embryo after ISH with *hoxa1 *and *otx2 *(red). **(N) ***gbx1*-injected embryo; the *hoxa1 *expression doamin is enlarged. **(O) **WT embryo after ISH with *cdx4 *and *otx2 *(red). **(P) ***gbx1*-injected embryo; the *cdx4 *expression domain is enlarged. **(Q) **WT embryo after ISH with *val *and *krox20 *(red). **(R) ***gbx1*-injected embryo; *val *expression is not affected: the *krox20 *expression domain corresponding to rh3 (green arrowhead) is enlarged, whereas rh5 is less affected. (A, B, E-P) Lateral views, anterior to the left; (C, D-Q, R) dorsal views, anterior to the top; (A'-L') flat-mounted embryos, anterior to the top.

**Figure 3:**
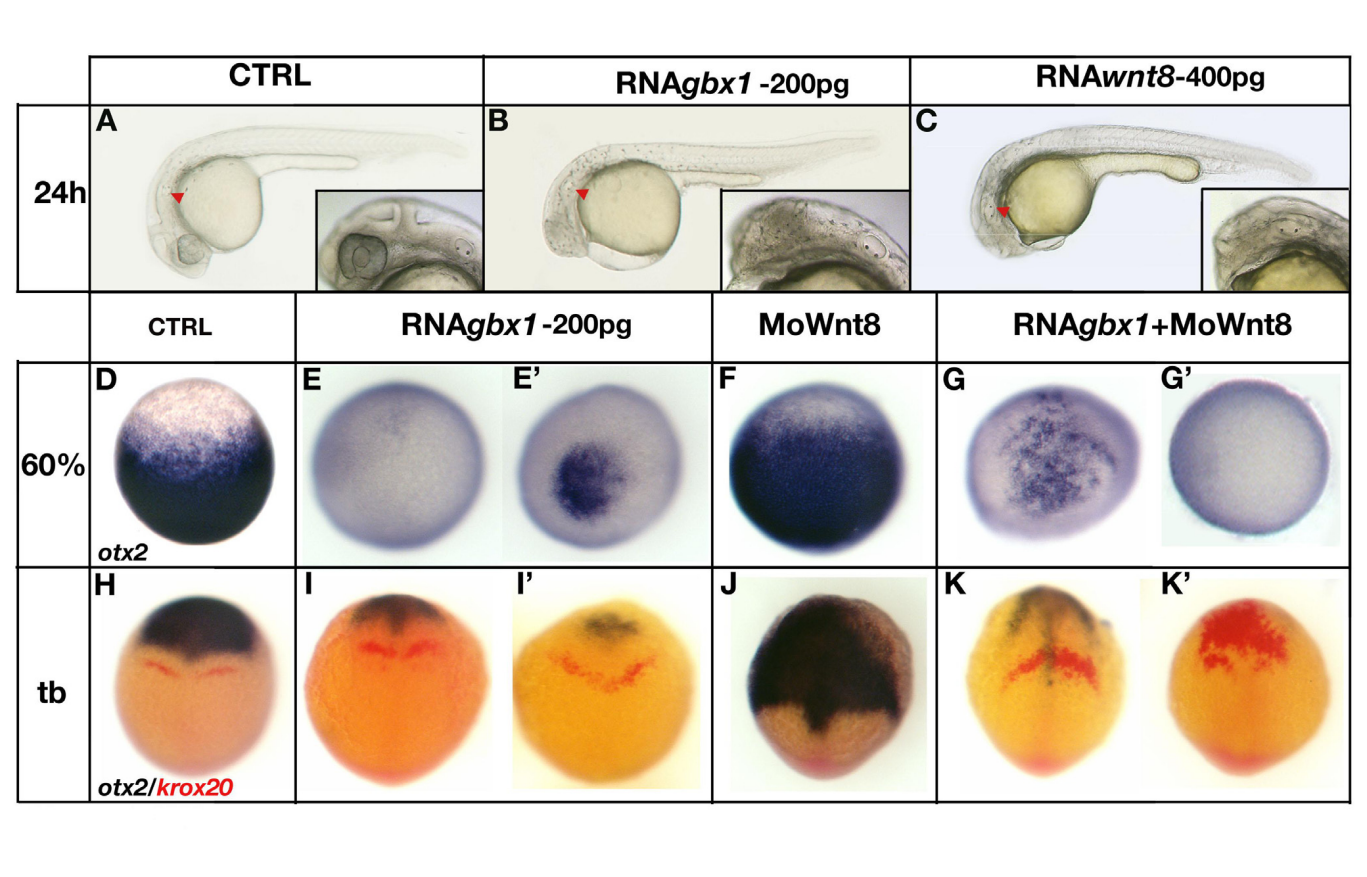
***gbx1 *acts as a mediator of the Wnt8 hindbrain posteriorizing signal**. **(A) **Control embryo at 24 h, **(B) ***gbx1*-injected embryo (200 pg), **(C) ***wnt8*-injected embryo (400 pg). *gbx1 *overexpression mimics *wnt8 *gain-of-function; the embryos are truncated anteriorly. The red arrowhead indicates the position of the ear. **(D-G') **Animal pole views at 60% epiboly after ISH with *otx2*. (D) Control embryo, (E, E') *gbx1*-injected embryos (200 pg), (F) embryo injected with *wnt8 *morpholinos, (G, G') embryos co-injected with *gbx1 *mRNA and *wnt8 *morpholinos. Expansion of the *otx2 *domain in the *wnt8 *morphant is rescued by co-injection with *gbx1 *mRNA. **(H-K') **Dorsal views at the tailbud (tb) stage after ISH with *otx2 *(blue) and *krox20 *(red). (H) Control embryo, (I, I') *gbx1*-injected embryos, (J) embryo injected with *wnt8 *morpholinos, (K, K') embryos co-injected with *gbx1 *mRNA and *wnt8 *morpholinos. Loss of *krox20 *and posterior expansion of the *otx2 *domain in the *wnt8 *morphant is rescued by *gbx1 *mRNA.

To study the effect of *gbx1 *overexpression during neural plate regionalization, we analyzed *gbx1*-injected embryos at the tailbud stage using *otx2 *as a forebrain/midbrain marker, and *krox20 *as a hindbrain marker specific for rhombomeres (rh) 3 and 5. Increasing amounts (40–500 pg) of injected *gbx1 *mRNA gradually removed all anterior neural fates (Figure 1G–J), with the highest concentrations resulting in rh3 occupying the rostral end of the embryo (Figure 1G–I'). In all cases, *otx2 *expression was strongly reduced or absent, whereas *krox20 *expression was expanded (Figure 1G–I'). This expansion was associated with rh3 rather than rh5 (Figures 1I' and 2Q, R), suggesting that rh3 is more sensitive to *gbx1*. The absence or reduction of *otx2 *was already observable at 60% epiboly (Figure 3E, E').

In sum, these data show that ectopic *gbx1 *represses *otx2 *expression, reduces tissue with forebrain/midbrain identity and increases the amount of tissue with posterior identity (hindbrain, ear). We conclude that *gbx1 *has posteriorizing activity at early stages of development, acting during hindbrain development and, as suggested by its repression of *otx2*, during demarcation of the MHB primordium.

### *gbx1 *induces posterior cell fate by transforming anterior neural tissue

**Figure 4:**
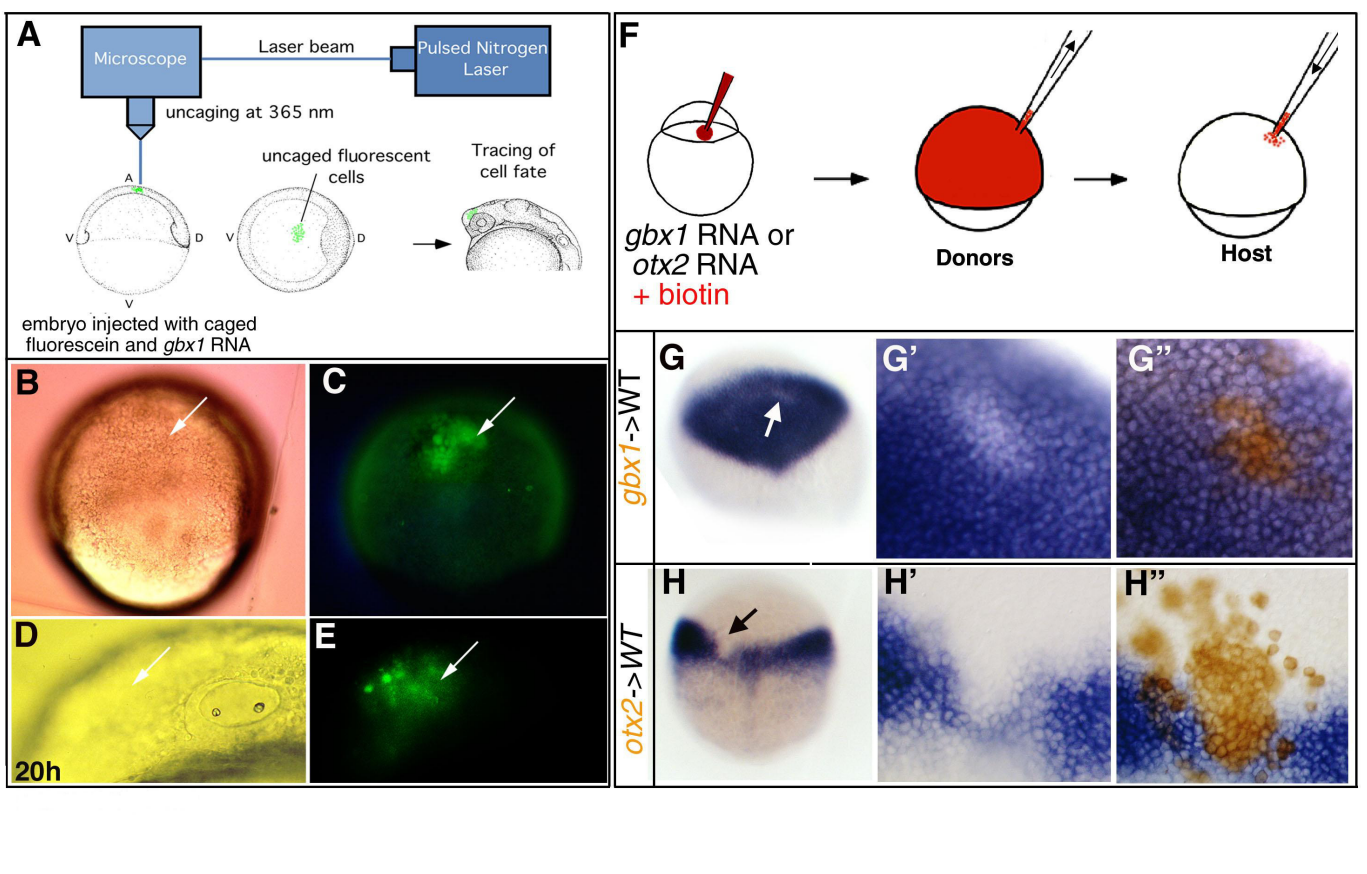
***gbx1 *induces posterior neural fate via transformation of anterior neural fate and *gbx1 *and *otx2 *repress each other cell-autonomously**. **(A) **Schematic drawing of the fluorescein uncaging procedure (for details, see [33]). **(B, C) ***gbx1*-injected embryo after uncaging cells in the prospective *otx2 *domain. **(D, E) **After 20 h of development the labeled cells come to lie in the anterior of the embryo (arrow). (B, D) Nomarski optics; (C, E) detection of the fluorescent cells (arrow). **(F) **Experimental procedure for cell transplantations. Donor embryos are generated by co-injecting biotinylated dextran as lineage tracer and *gbx1 *(300 pg) or *otx2 *mRNA (300 pg). Cells are taken out of the donor at 40% epiboly and transplanted into a wild-type (WT) host embryo. **(G) **Chimeric embryo containing cells derived from embryos injected with biotinylated dextran and *gbx1*mRNA, and stained for *otx2 *(blue). Unlabeled patch of cells marked by a white arrow. **(G') **Close-up of the patch indicated in (G) before biotin staining and **(G") **after biotin staining (brown). *gbx1 *overexpressing cells within the *otx2 *domain do not express *otx2*. **(H) **Chimeric embryos containing cells derived from embryos injected with biotinylated dextran and *otx2 *mRNA, and stained for *gbx1 *(blue). Unlabeled patch of cells marked by a black arrow. **(H') **Close-up of the patch indicated in (H) before biotin staining and **(H") **after biotin staining (brown). *otx2 *overexpressing cells within the *gbx1 *domain do not express *gbx1*.

The observed loss of anterior neural tissue and expansion of posterior neural tissue in *gbx1*-injected embryos could result from either transformation of anterior tissue towards a posterior fate, or a loss of anterior tissue due to cell death. To distinguish between these possibilities, we used a labeling technique to follow small cell clones [33] in the presumptive midbrain neural plate of *gbx1*-misexpressing embryos, and to map their location at 20 h of development. Embryos were injected at the one-cell stage with a caged-fluorescent dye that was later uncaged in a small group of cells at the appropriate stage by a nitrogen laser emitting a wavelength of 365 nm (Figure 4A). In embryos co-injected with 200 pg *gbx1*-mRNA, we activated fluorescein at 60% epiboly in a group of cells within the prospective *otx2 *domain as determined by the established fate map [34]) (Figure 4B, C). The fate of these fluorescent cells was then followed throughout development. At 20 h, the labeled cells sat in the rostral part of the embryo, close to the otic vesicle (Figure 4D, E), suggesting that although these cells were fated to become anterior, *gbx1 *overexpression transformed them into a posterior identity. In addition, TUNEL analysis of *gbx1*-injected embryos at the tailbud stage showed no difference in the distribution of apoptotic cells compared to control embryos, suggesting that there is no increase in cell death (data not shown). We therefore conclude that *gbx1 *is capable of repressing midbrain/forebrain identity within the neuroectoderm, most likely expanding posterior neural tissue via the transformation of anterior neural fates. This supports a role for *gbx1 *in specifying hindbrain cell fate.

### *gbx1 *and *otx2 *repress each other cell-autonomously

The observed repressive action of *gbx1 *led us to ask whether *gbx1 *acts cell-autonomously to repress *otx2*. To address this, *gbx1*-overexpressing cells (300 pg) were transplanted into the prospective *otx2 *domain of wild-type embryos at 50% epiboly, and assayed for *otx2 *expression at 80% epiboly (Figure 4F–G"). Transplanted *gbx1*-positive cells that came to lie within the *otx2 *expression domain did not express *otx2*, whereas all adjacent *gbx1*-negative cells were *otx2 *positive (Figure 4G–G"; n = 15). This indicates that the absence of *otx2 *expression in *gbx1*-expressing cells is a cell-autonomous effect.

As mutual repressive interactions may exist between *Otx *and *Gbx *genes (see Introduction; reviewed in [10,11,26]), we next asked if *otx2 *can repress *gbx1 *expression in a cell-autonomous manner. For this purpose, we ubiquitously overexpressed *otx2 *by injecting mRNA in one-cell stage embryos. Although these embryos showed aberrant epiboly and gastrulation movements (data not shown) due to the action of *otx *gene products in embryonic cell aggregation [35,36], we could observe that *gbx1 *expression was strongly diminished (data not shown). To evaluate the interaction between *otx2 *and *gbx1 *in the context of a normal gastrulating embryo, we transplanted *otx2*-overexpressing cells (300 pg) at 50% epiboly into the prospective *gbx1 *domain of wild-type embryos and assayed for *gbx1 *expression at the tailbud stage (Figure 4F, H–H"). The transplanted *otx2*-positive cells that came to lie within the *gbx1 *expression domain did not express *gbx1*, but all adjacent *otx2*-negative cells were *gbx1 *positive (Figure 4H–H"; n = 10). This result suggests that the repression of *gbx1 *in *otx2*-expressing cells is a cell-autonomous effect.

Altogether, these results show that zebrafish *gbx1 *and *otx2 *can mutually repress each other in an autonomous manner. This supports the idea that, as described in the mouse for *Gbx2 *and *Otx2*, such mutual exclusion may be a mechanism to subdivide the neural plate into juxtaposed *otx *and *gbx *expression domains that will be required to position the MHB.

### *gbx1 *overexpression acts in a concentration-dependant manner to transform midbrain and forebrain territories

In the above experiments, we found that increasing amounts of injected *gbx1 *mRNA gradually removed all anterior neural fates: intermediate concentrations reduced the *otx2 *expression domain (Figure 1H, H') and higher concentrations completely abolished it (Figure 1I–I'). This suggested that not all regions of the *otx2 *domain are equally sensitive to *gbx1*, perhaps because of differences between fore- and midbrain response.

To investigate this possibility, we injected intermediate doses of *gbx1 *mRNA in order to retain residual *otx2 *expression (Figure 1H–H'), and followed the identity of this *otx2 *domain by analyzing fore- and fore/midbrain specific marker expression using dual labeling. Analysis of the forebrain marker *six3 *showed that the majority of the residual *otx2 *domain is *six3*-positive, leaving only a few cells that express *otx2 *alone (Figure 2A–B'). This suggests that the *otx2*-positive territory in *gbx1*-injected embryos largely possesses a forebrain identity and that the midbrain identity is almost completely lost. We also found that another forebrain marker, *emx1*, was partly reduced (Figure 2C–D'). Noticeably, two bilateral transverse *emx1 *stripes were still visible in the posterior forebrain (diencephalic primordium) [37], suggesting that the posterior forebrain is present.

Together, these findings indicate that ectopic *gbx1 *at relatively low doses leads to local transformation that affects the midbrain, the territory adjacent to its normal expression domain; only high doses of *gbx1 *transform forebrain into more posterior neural fates. These data suggest differences in fore- and midbrain response to *gbx*1 and further support a role for *gbx1 *in positioning the *otx2 *posterior border at which the MHB primordium forms.

### *gbx1 *overexpression shifts the midbrain-hindbrain boundary anteriorly

Previous analysis of *gbx1 *expression in wild type and in *noi*/*pax2.1*, *spg*/*pou2 *and *ace*/*fgf8 *mutants suggested that *gbx1 *might act upstream of these genes as an important regulator of MHB formation, particularly during the maintenance phase [8,38]. Therefore, the effect of *gbx1 *overexpression on these MHB markers was examined. Analysis of *pax2.1*, which is expressed at the tailbud stage in the posterior midbrain and the anterior hindbrain [8,13], and *fgf8*, expressed in the anterior hindbrain with its anterior border abutting the *otx2 *expression domain [39], showed that these genes were activated ectopically in the anterior neural plate when *gbx1 *was overexpressed (Figure 2E–H'). This phenotype was observed with high frequency when 200 pg of *gbx1 *RNA was injected. In the *gbx1*-injected embryos, the *pax2.1 *mid-hindbrain expression domain shifted anteriorly as the distance between this domain and the lateral plate mesoderm expressing domain increased (compare the black bars in Figure 2E, F). Furthermore, the anterior limit of the *fgf8 *expression domain, which corresponds to the MHB, also shifted anteriorly as the distance between this domain and Fgf8-expressing cells at the margin increased (Figure 2G, H). *pax2.1 *and *fgf8 *expression was also analyzed after dual labeling with an *otx2 *probe (red), which showed that *pax2.1 *and *fgf8 *domains retain a normal spatial relationship with respect to the *otx2 *posterior border (Figure 2E'–H', green arrowheads). The overall anterior displacement of the *otx2*/*pax2.1 *and *otx2*/*fgf8 *domains further shows that ectopic *gbx1 *expression is able to relocate the MHB.

*wnt1 *and *eng2 *also belong to the set of genes expressed at the *gbx1*/*otx2 *interface and required for MHB organizer maintenance. *wnt1 *is expressed at the tailbud stage in the posterior midbrain where it overlays the posterior border of *otx2 *[40], and *eng2 *is expressed in the posterior midbrain and anterior hindbrain [41,42]. In contrast to *fgf8 *and *pax2.1*, *wnt1 *and *eng2 *were not ectopically induced in *gbx1*-injected embyos (Figure 2I–L'). Both genes' expression domains were reduced in the gbx1-overexpressing embryos. Co-labeling with *otx2 *showed that their residual expression was located anteriorly within the neural plate, retaining a normal spatial relationship with the *otx2 *posterior border (Figure 2I'–L", green arrowheads) and marking the abnormally anteriorly positioned MHB.

Our results demonstrate that changing the A-P position of the *gbx1*/*otx2 *interface, by ectopically expressing *gbx1*, consistently changes the position of early MHB marker onset (*fgf8*, *pax2*, *1*, *wnt1 *and *eng2*) and, thus, the location of the developing MHB organizer.

### *gbx1 *overexpression mainly expands the anterior hindbrain territory

Previous analysis of *gbx1 *expression showed that it is also expressed in the hindbrain and spinal cord primordium from 60% epiboly onwards [8]. Morphological (not shown) and *in situ *hybridization (ISH) analysis of *krox20 *in *gbx1*-injected embryos (Figure 1) revealed that the posterior part of the embryo was enlarged, prompting us to further study hindbrain development. For this we looked at *hoxa1*, which is expressed in the hindbrain with an anterior limit corresponding to the border rh3/rh4 [43], *valentino *(*val*), which is expressed in rh5 and rh6 [44], and a *caudal*-related gene, *cdx4 *[45], which is expressed in the posterior third of the embryo[45]. In *gbx1*-injected embryos, the *hoxa1 *expression domain is strongly expanded (Figure 2M, N), whereas the *val *expression domain remains stable (Figure 2Q, R). Interestingly, in the same embryo *krox20 *expression in rh3 (red) is expanded (Figure 2Q, R). This shows that all rostral boundaries up to rh3 are moved rostrally and keep their relative position to each other. The posterior hindbrain (*val *domain) seems less sensitive to *gbx1 *overexpression. *cdx4 *expression domains is strongly expanded (Figure 2O, P), supporting an enlargement of the prospective spinal cord territory.

### *gbx1 *mediates the Wnt8 hindbrain posteriorizing signal

Previous studies have shown that Wnt8, a posteriorizing factor, acts upstream of *gbx1 *[32], and that Wnt8 loss-of-function strongly impairs hindbrain and spinal cord development [46-48]. Interestingly, like *gbx1*, ectopic *wnt8 *activation is known to expand posterior neural fates, resulting in anterior head truncation (Figure 3C) [46,48,49]. We found that gain-of-function of either gene truncated the entire forebrain/midbrain (Figure 3A–C), while expanding the hindbrain and shifting the otic vesicle anteriorly (Figure 3A–C, red arrowheads). If Wnt8 induction of *gbx1 *expression is instrumental in mediating Wnt signaling effects on neuroectoderm patterning, the loss of Wnt8 may be rescued by restoring *gbx1 *function. To test this model, we investigated if *gbx1 *can rescue the loss of posterior tissue in *wnt8 *morphants by co-injecting wild-type embryos with *gbx1 *mRNA (200 pg) and *wnt8 *antisense morpholinos [32,48]. As previously described, *wnt8*-morphants display a ventro-laterally expanded *otx2 *expression domain (Figure 3F, dorsal views) [32,48], and *gbx1*-injected embryos lack almost all *otx2 *at 60% epiboly (Figure 3E, E'). Co-injection of *wnt8 *morpholinos and *gbx1 *mRNA resulted in a loss of *otx2 *expression (50%, n = 20) or a faint patchy expression (50%, n = 20) (Figure 3G, G'), suggesting that *gbx1 *reduced or abolished the *otx2 *expansion observed in the *wnt8 *morphants. Similar effects on *otx2 *levels were observed at the tailbud stage (Figure 3H–K', blue labeling). At this stage, loss of *krox20 *expression was also seen in *wnt8 *morphants (65%, n = 50; Figure 3J). Co-injection with *gbx1 *mRNA rescued this *krox20 *domain, which, however, was mislocated at the A-P level (90%, n = 40; Figure 3K, K').

Together, these findings demonstrate that *gbx1 *can partially restore hindbrain patterning in the absence of Wnt8, suggesting that *gbx1 *compensates for loss of Wnt8. Considering that Wnt8 is required for the onset of *gbx1 *expression, as well as for the correct A-P positioning of its expression domain [32], we propose that *gbx1 *acts at the transcriptional level to mediate Wnt8 posteriorizing effects on hindbrain patterning. In view of the large hindbrain *gbx1 *expression domain [8], this *gbx1 *function in hindbrain patterning could be independent of its role in MHB positioning.

### *gbx1 *loss-of-function reduces the antero-posterior extent of the hindbrain/spinal cord territory

We next investigated the effect of *gbx1 *loss-of-function on zebrafish development by inhibiting mRNA translation with antisense morpholinos [50]. Two non-overlapping morpholinos, Mo1 and Mo2, were designed in the 5' untranslated region of *gbx1*. Injecting either of these morpholinos produced similar morphological phenotypes (Additional file 1A–F). When Mo1 was injected at a low dose (1–5 ng), the embryos developed properly, displaying only weak MHB abnormalities at 24 h. At an elevated dose (5–10 ng), embryonic development slowed during gastrulation such that, from the tailbud stage, the injected embryos failed to elongate properly (Additional file 1D, E). At 24 h, the anterior brain region of the injected embryos was highly affected and the A-P extent of the hindbrain was reduced (Additional file 1D, E). Mo2 generated the same phenotypes already at lower doses (Additional file 1D, F)

**Figure 5:**
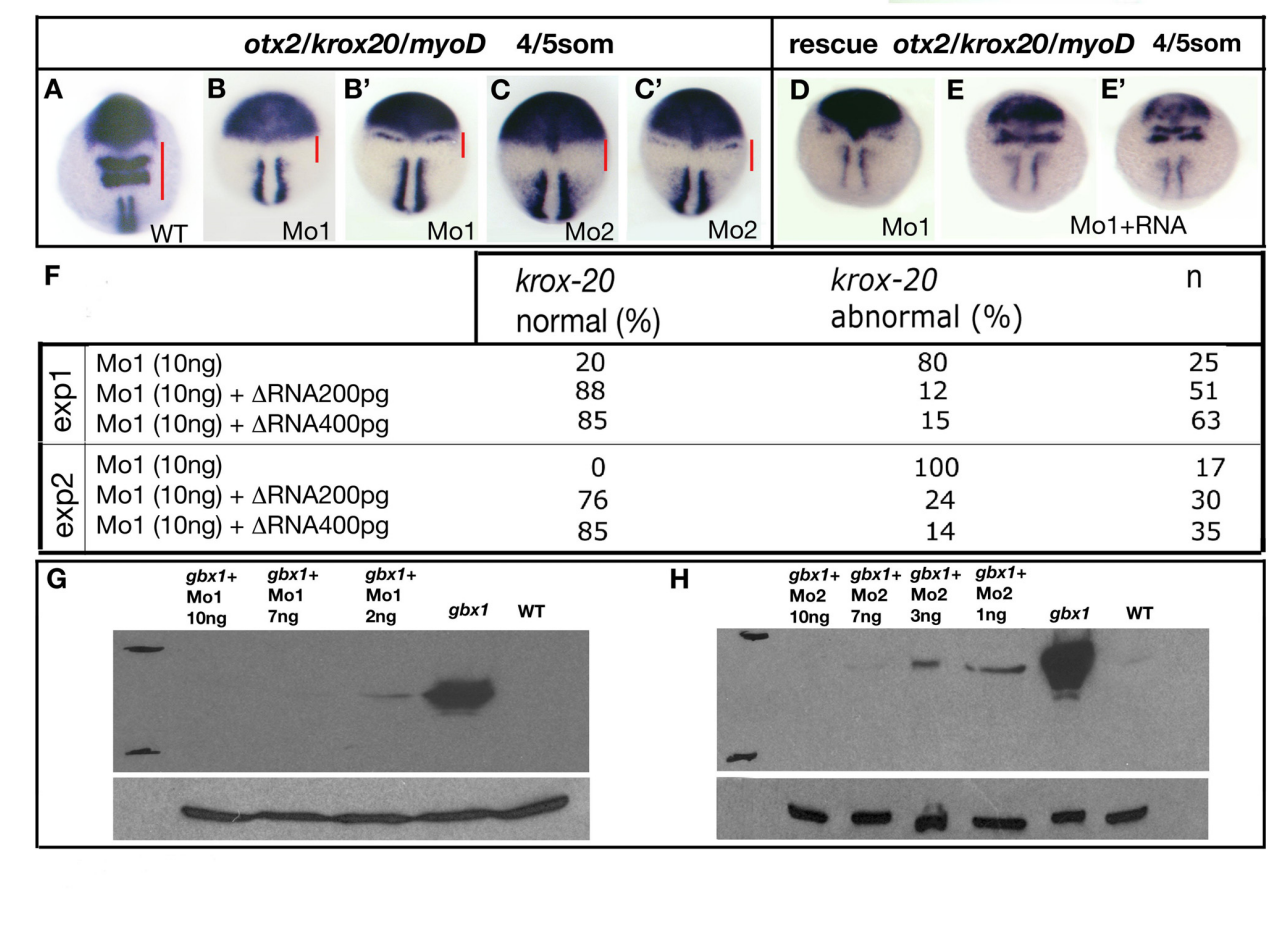
***gbx1 *morpholinos affect hindbrain patterning**. **(A-C') **Embryos at the four somite stage stained for *otx2*/*krox20*/*myod*. (A) Control embryo, (B, B') embryos injected with 10 ng Mo1, (C, C') embryos injected with 7 ng Mo2. Injections of two different morpholinos show the same phenotype morphologically and after *in situ *hybridization analysis. **(D-F) **Rescue experiments using the *krox20 *expression domain as a read-out. (D) *gbx1*-morphant, (E, E') rescued morphant embryos co-injected with *gbx1 *mRNA that does not contain the MO binding sequence in its 5' region. (F) Two combinations were tested: 10 ng of Mo1 with 200 pg or 400 pg of *gbx1 *mRNA. **(G, H) **Western blot detection showing down regulation of *gbx1-myc *mRNA translation in embryos co-injected with *gbx1-myc *mRNA containing morpholino binding sequences and Mo1 (G) or Mo2 (H). WT, wild type.

Considering that *gbx1 *is initially expressed in the anterior hindbrain and spinal cord primordium, we first investigated whether these territories are affected in embryos lacking *gbx1 *protein. We injected morpholinos at concentrations that maximally blocked translation (10 ng of Mo1, 5 ng of Mo2; Figure 5G, H). Because the *gbx1 *morphants developed large amounts of necrosis at 20–24 h (Additional file 1D–F), we focused our analysis on stages from gastrulation to five somites. We used *krox20 *to mark rh3 and rh5, and *otx2 *to mark the forebrain/midbrain. Embryos were co-labeled with *myoD*, a basic helix-loop-helix (bHLH) transcription factor expressed in adaxial mesoderm and the forming somites, allowing us to properly stage the embryos. In Mo1-injected embryos stained for *otx2*/*krox20*/*myoD *at the four-somite stage, we found that the distance between the posterior border of *otx2 *and the rostral limit of *myoD *was smaller along the A-P axis (Figure 5A–B', red bars). Also, the overall length of the *myoD *expression domain was shorter, suggesting a reduction in spinal cord length. Injection of Mo1 or Mo2 led to a complete loss of *krox20 *in 50% of the embryos (Figure 5B, C). In the other 50%, only one stripe was present and its width was reduced in comparison with the wild-type *krox20 *stripes (Figure 5A–C'). The use of *myoD *to stage the embryos, allowing the somites to be counted, confirms that the presence of only one *krox20 *stripe is not the result of delayed development, and further analysis (see below) confirmed the rh3 stripe is missing (Additional file 2). Overall, these data indicate that loss of *gbx1 *affects the anterior hindbrain and spinal cord.

Our next step was to validate *gbx1 *morpholino specificity by performing two sets of experiments. First, co-injection of individual morpholinos and *gbx1-myc *tagged mRNA containing sequences for morpholino binding was followed by protein extraction and western blot detection of the myc epitope, which confirmed that both Mo1 and Mo2 efficiently block translation of *gbx1 *mRNA (Figure 5G, H). Because more Mo2 than Mo1 had to be injected to obtain the same degree of inhibition, and Mo2 induced high levels of general necrosis, Mo1 was utilized for all further experiments. In our second test of morpholino specificity, we attempted to rescue morphants with a synthetic *gbx1 *mRNA that cannot be bound by the morpholino (Dgbx1), and analyzed *krox20 *domains as a read-out (Figure 5D–F). Two combinations were used: 10 ng of Mo1 plus either 200 pg or 400 pg of Dgbx1 mRNA. As shown in Figure 5F, co-injected embryos expressed *krox20 *in two distinct stripes, whereas almost no expression was seen in embryos injected with Mo1 alone (Figure 5F). This rescue by Dgbx1 RNA suggests that the loss of *krox20 *expression is specific to the loss of *gbx1 *protein, rather to secondary effects of the morpholino. Not surprisingly, the observed necrosis at 24 h could not be rescued by co-injection with Dgbx1 mRNA, indicating that this phenotype could be attributed to secondary morpholino effects.

Our combined results therefore support the specificity of the *gbx1 *morpholino phenotype: first, identical phenotypes were observed with two different morpholinos (Figure 5A–C'); second, western blot analysis demonstrated that both Mo1 and Mo2 block translation of *gbx1-myc *mRNA (Figure 5G, H); third, we could rescue loss of *krox20 *expression using *gbx1 *mRNA (Figure 5D–F).

**Figure 6:**
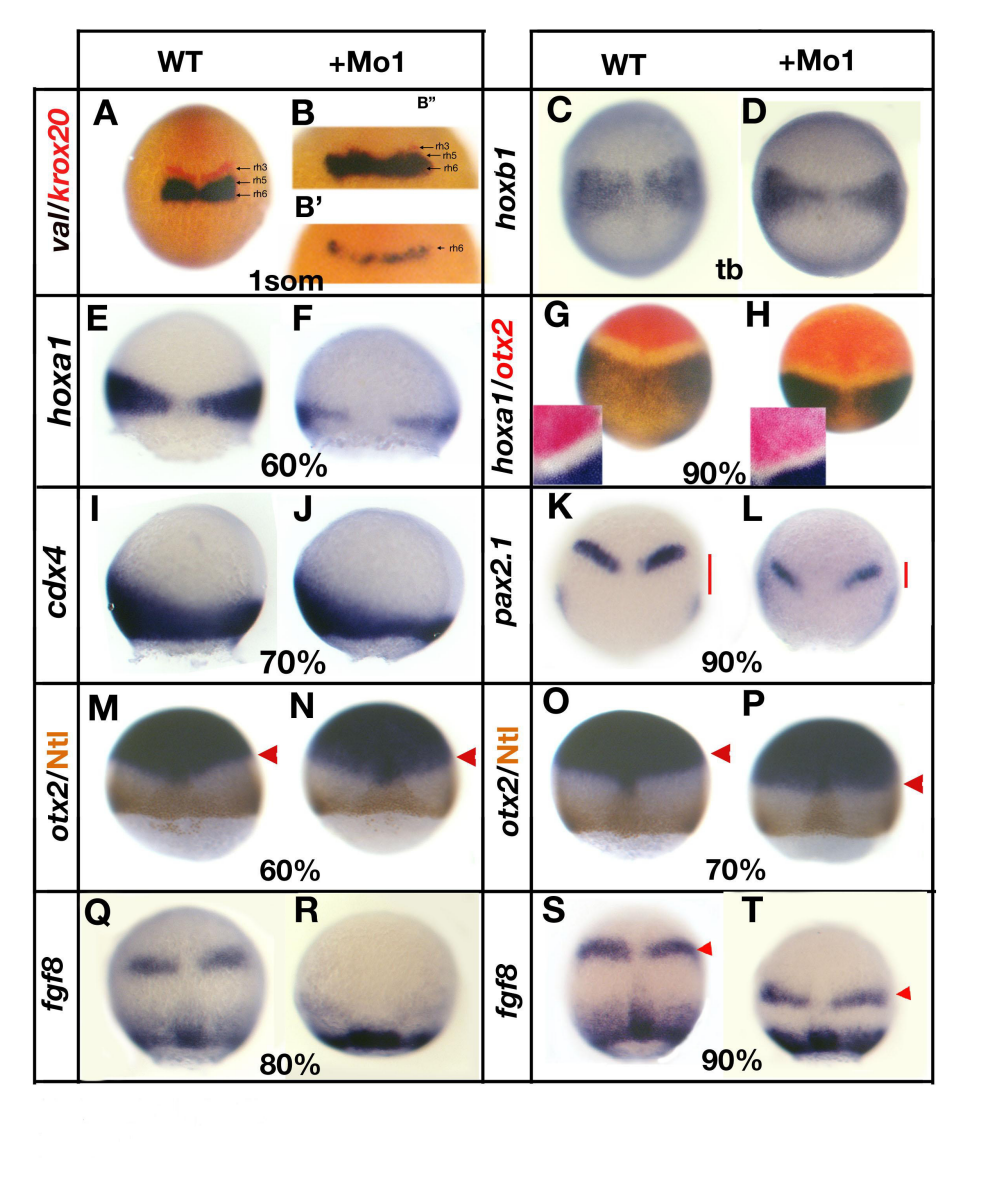
**Loss of *gbx1 *affects hindbrain and midbrain-hindbrain boundary (MHB) markers**. **(A-H, M-K-T) **are dorsal views, **(I, J) **are lateral views and the developmental stages are indicated (tb, tailbud stage). (A) Control embryo (wild type (WT)) stained with *val *(blue) and *krox20 *(red), (B, B') *gbx1*-morphants. A residual *krox20 *expression is seen in (B') and no expression of *krox20 *in (B"). For (B', B") a higher magnification of the region of interest is shown. (C) Control embryo stained with *hoxb1*; (D) *gbx1*-morphant. (E) Control embryo stained with *hoxa1*; (F) *gbx1*-morphant. (G) Control embryo stained with *hoxa1 *and *otx2*; (H) *gbx1*-morphant. A higher magnification is shown, illustrating the reduction of size of the gap between the *otx2 *and *hoxa1 *domains. (I) Control embryo stained with *cdx4*; (J) *gbx1 *morphant. (K) Control embryo stained with *pax2.1*; (L) *gbx1*-morphant. The red bars show the distance between the MHB and the lateral plate mesoderm expression domains. This distance is markedly reduced in the *gbx1 *morphants. (M, O) Control embryos stained with *otx2 *(blue) and Ntl antibody (brown); (N, P) *gbx1 *morphants. The *otx2 *domain is unaffected at 60% epiboly but slightly shifted posteriorly at 70% (red arrowheads). (Q, S) Control embryos stained with *fgf8*; (R, T) *gbx1*-morphants. MHB expression is delayed (R) and shifted posteriorly (S, T, red arrowheads) in the morphants.

The loss of *krox20 *expression in the hindbrain of embryos injected with *gbx1 *morpholino suggests that some rhombomeres are not specified properly. To investigate this we looked at *valentino *(*val*), a marker of rh5 and rh6. In 50% of *gbx1 *morphants, we observed the two stripes corresponding to *val *expression in rh5 and rh6 (Figure 6A, B, B'). Co-staining with *krox20 *shows a faint red band anteriorly to the two blue stripes corresponding to the remaining expression of *krox20 *in rh3 (Figure 6A, B, B'). In the other 50% of *gbx1 *morphants, only one stripe of *val *was observed and co-staining with *krox20 *indicated that the remaining stripe corresponds to rh6 (Figure 6B"). Thus, we suggest that *krox20 *expression is lost in rh3 and that only in the most affected morphants is its expression is lost in rh5. This corroborates our gain-of-function experiments where we observed that rh3 is more sensitive to *gbx1*. We then analyzed expression of several hindbrain markers in *gbx1 *morphants at 60–90% epiboly. We found reduced *hoxb1 *expression in rh4 and spinal cord, suggestive of a shortened hindbrain/spinal cord domain (Figure 6C, D). Expression of *hoxa1 *at 60% epiboly (Figure 6E, F) and the tailbud stage (Figure 6G, H), and *cdx4 *at 70% epiboly (Figure 6I, J), also indicated hindbrain reduction. Furthermore, the size of the gap between *otx2 *and *hoxa1 *expression domains was reduced (Figure 6G, H), suggesting that *gbx1 *is required locally for hindbrain specification.

Altogether, the analysis of various markers in *gbx1*-morphants reveals a shortening of the hindbrain and spinal cord that suggests a reduction in posterior neural fates. These observations complement our gain-of-function results, thereby supporting a role for *gbx1 *in mediating posteriorization signals that pattern the hindbrain.

### *gbx1 *loss-of-function shifts the midbrain-hindbrain boundary posteriorly

Having found that *gbx1 *overexpression relocates the MHB primordium more anteriorly, we next sought to assess early neural plate development in *gbx1 *morphants. Examination of the *otx2 *domain together with Ntl antibody staining to visualize the margin of the embryo in the *gbx1 *morphants showed no abnormalities at 60% epiboly (Figure 6M, N). However, starting at 70% epiboly, a slight caudal shift of the posterior *otx2 *border was observed (Figure 6O, P). We have previously shown that the *gbx1 *and *otx2 *expression domains overlap by three to four cell rows at 60%; however, at 70% the two domains abut each other without overlap [8]. This suggests that *gbx1 *is required to correctly establish the *otx2 *posterior border starting at 70% epiboly. Additionally, it indicates that there is a phase when the *otx2 *and *gbx1 *domains are established independently of each other before they interact.

To determine the role of *gbx1 *in MHB development, we examined the effect of *gbx1 *loss-of-function on MHB gene expression. *fgf8 *(Figure 6Q–T), *pax2.1 *(Figure 6K, L), *wnt1 *and *eng2 *(not shown) were all expressed in *gbx1*-morphants, although *fgf8 *was slightly delayed (Figure 6Q, R). This suggests that Gbx1 protein is not required for initial MHB marker expression and, by extension, for the establishment of MHB organizing activity. The presence of MHB gene expression also indicates that the maintenance regulatory loop functions correctly in the absence of Gbx1. Nonetheless, the A-P position of *fgf8 *(Figure 6S, T) and *pax2.1 *(Figure 6K, L) MHB domains was posteriorly shifted in *gbx1 *morphants. At 80% epiboly, morpholino injections led to a reduction in the A-P distance between *fgf8 *expression domains in the MHB primordium and the margin (Figure 6S, T, red arrowheads). Similarly, in *gbx1 *morphants at 80% epiboly, the distance between *pax2.1 *domains in the MHB primordium and the lateral plate mesoderm was decreased (Figure 6K, L, red bars) and the expression was reduced in both domains. Thus, our results indicate that in the absence of Gbx1 protein, the *otx2 *expression domain shifts slightly to the posterior and leads to a similar displacement of the MHB as reflected by shifts in *fgf8 *and *pax2.1*.

### Juxtaposition of *gbx1 *and *otx2 *can induce MHB markers

**Figure 7:**
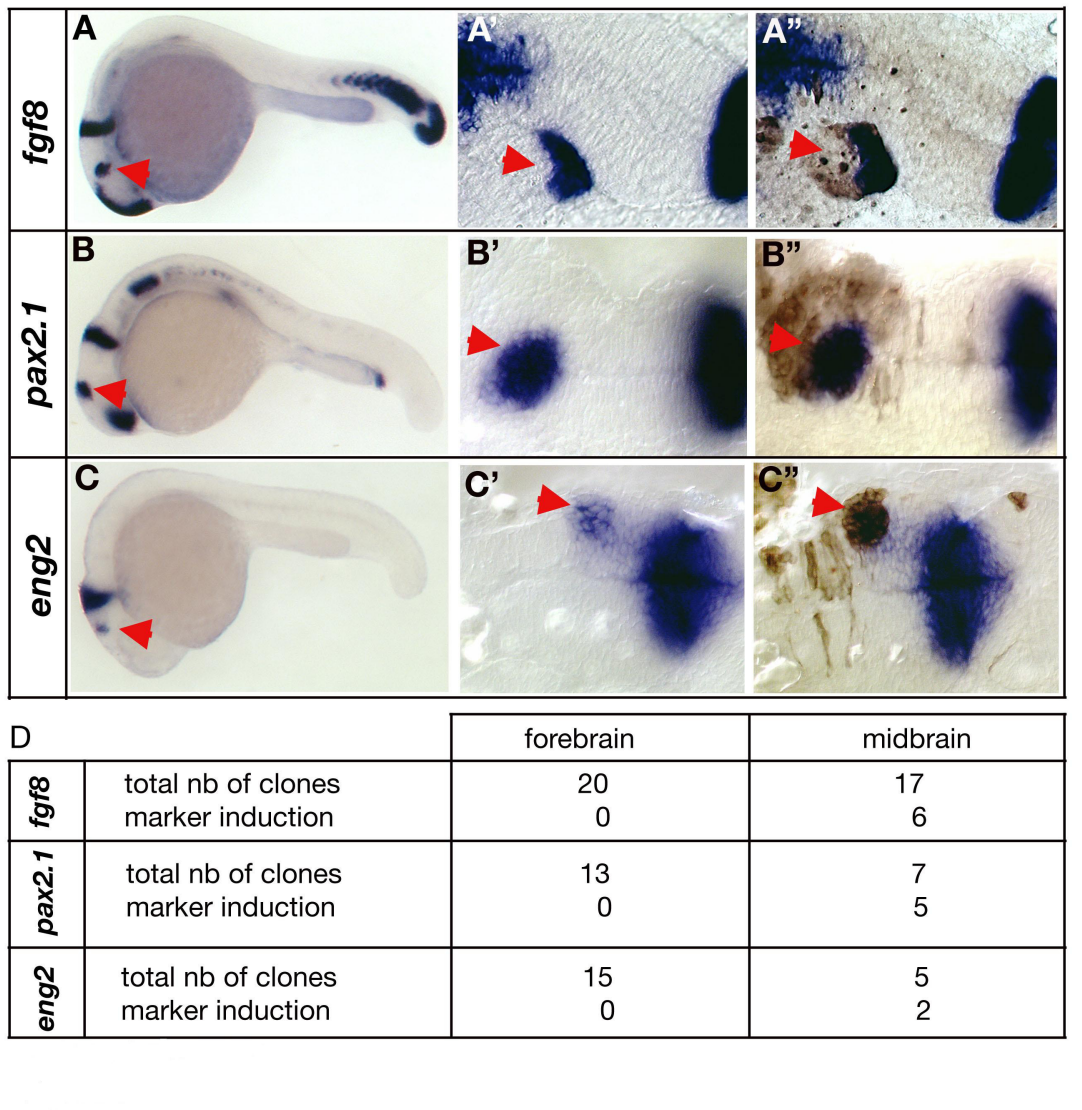
**The *otx2*/*gbx1 *interface plays an essential role in midbrain-hindbrain boundary development**. **(A) **Lateral view of a chimeric embryo stained for *fgf8*. The red arrowhead indicates ectopic *fgf8 *expression. **(B) **Lateral view of a chimeric embryo stained for *pax2.1*. The red arrowhead indicates ectopic *pax2.1 *expression. **(C) **Lateral view of a chimeric embryo stained for *eng2*. The red arrowhead indicates ectopic *eng2 *expression. **(A', B', C') **Close-up of the ectopic patch before biotin staining and **(A", B", C") **after biotin staining (brown). For all the three genes, ectopic expression is induced in *gbx1*-overexpressing cells within the *otx2 *domain (red arrowheads). **(D) **Summary table of the transplanted cells' positions and marker induction. Clones localized in the forebrain never induced ectopic *fgf8*, *pax2.1 *or *eng2 *expression.

Our data reveal that an interaction between *otx2 *and *gbx1 *defines the position of the MHB organizer in the early neural plate. Given this, ectopic juxtaposition of an *otx2 *and *gbx1 *domain should trigger the expression of MHB markers. To test this hypothesis, we transplanted cells from *gbx1*-injected embryos (500 pg) into the *otx2 *domain of wild-type embryos to generate an ectopic *otx2*/*gbx1 *interface, and then looked at markers for MHB induction. We found that three MHB markers, *fgf8*, *pax2.1 *and *eng2*, were induced at this ectopic interface at 24 h of development (Figure 7). Induction of these markers was preferentially observed in the more posterior *otx2 *domain (Figure 7A–C", arrowheads) and correlated with the size of the clone, as small clones or single cells did not induce ectopic MHB marker expression. We also observed that clones localized in the forebrain territory never induced MHB markers (Figure 7D). It was previously found that neither isthmus grafts nor Fgf8 beads placed in prosomere 2 (diencephalon) induce host tissue to form cerebellum [51,52]. Our observations agree with this finding in suggesting that additional factors expressed at the MHB level are required together with *otx2 *and *gbx1 *to initiate MHB marker expression.

As a whole, these results show that a MHB molecular cascade, including *fgf8*, *pax2.1 *and *eng2*, is selectively triggered at the point of ectopic juxtaposition of *otx2 *and *gbx1 *domains in a competent neuroepithelium in zebrafish.

## Discussion

Previous studies have shown that in amphibian and amniotes (chick, mouse, *Xenopus*), mutual repression between *Otx2 *and *Gbx2 *at the future MHB results in the positioning and sharpening of the *Otx2*/*Gbx2 *border (reviewed in [10,26]). Similar to *Gbx2 *expression in mouse [7,29], one of the zebrafish *gbx *genes, *gbx1*, is expressed adjacent to the *otx2 *domain at the end of gastrulation [8]. This led us to test if *gbx1 *plays a role in early neural patterning. Here we show that *gbx1 *does indeed play an important role in conjunction with *otx2 *to establish the MHB. Additionally, we describe a novel role for *gbx1 *as a mediator of the Wnt8 posteriorization signal required for hindbrain development. Thus, our work implicates *gbx1 *in two different aspects of early neural plate patterning.

### *gbx1 *represses *otx2 *expression and positions the MHB organizer

Our functional analysis supports the hypothesis that mutual repression between *gbx1 *and *otx2 *serves as a mechanism for MHB positioning. Ectopic *gbx1 *represses *otx2 *expression and repositions the MHB at the new *gbx1*/*otx2 *border. This is evidenced by an anterior shift in MHB marker gene expression, including the ectopic induction of *pax2.1 *and *fgf8 *in the anterior brain. Conversely, *gbx1 *loss-of-function allows the posterior expansion of the *otx2 *expression domain and repositions the MHB posteriorly. Considering our finding that *pax2.1*, *fgf8*, *eng2 *and *wnt1 *are expressed in *gbx1 *morphants, we suggest that, as in mouse [15-19], the activation and maintenance of MHB gene expression is independent of both *otx *and *gbx1 *function. Interestingly, although they are mislocated in *gbx1 *morphants, the *fgf8 *and *pax2.1 *domains are not expanded in *gbx1 *morphants and retain their shape. This could be due to *gbx2 *functioning later in development [8] to repress *otx2 *at its most posterior position and thereby keep its posterior border sharp.

Our gain-of-function experiments support the idea that *gbx1 *is involved in activation of *pax2.1 *and *fgf8*; however, it is clear from other work that *gbx1 *cannot directly activate *pax2.1 *expression. We previously observed that onset of *pax2.1 *expression occurs outside the endogenous *gbx1 *domain [8], and most likely requires an additional, diffusible signal. Co-injection of *gbx1 *and the Wnt inhibitor *dickkopf-1 *(*dkk1*), followed by staining for *pax2.1 *expression at 80% epiboly, showed that *pax2.1 *is not activated (MR and MB, unpublished results), suggesting that Wnt signals may be involved in the activation of *pax2.1*. Such signals could be *wnt8b*, *wnt1 *and/or *wnt10b *[46,53], all of which are expressed just before or during the onset of *pax2.1 *expression at the MHB. One explanation for ectopic *pax2.1 *activation in *gbx1 *overexpressing embryos is the fact that all A-P information, including *pax2.1 *activation signals, is shifted to more anterior regions.

Contrary to a general effect on MHB genes, *wnt1 *and *eng2 *are not ectopically expressed in *gbx1*-overexpressing embryos, but are instead downregulated. The failure of *gbx1 *to induce ectopic *eng2 *expression was indeed surprising considering that an earlier analysis of *noi*/*pax2.1 *mutants has shown that *pax2.1 *is necessary for *eng2 *activation [13]. It is possible that additional (co)factors, not induced in the *gbx1*-overexpressing embryos, might be involved. Indeed, it has been shown that *Xenopus engrailed-2 *is a direct target of Wnts [54], hinting at a link between *wnt1 *and *eng2 *diminished expression. Downregulation of *wnt1 *in *gbx1*-injected embryos is most likely the consequence of *otx2 *repression. In mouse, it has been shown that *Otx2 *is required cell autonomously for *Wnt1 *activation [15] and our data in zebrafish show that this pathway may be maintained, although it cannot be excluded that *gbx1 *directly represses *wnt1 *expression.

### *otx2 *and *gbx1 *interact to refine their respective expression domains

Our *gbx1 *loss-of-function data show that the *otx2 *domain remains robust at 60% epiboly but shifts slightly posterior at 70% epiboly. This is consistent with *otx2 *and *gbx1 *domains being independently established and interacting later to refine their respective expression domains. These data correlate with the observation in mouse that the presence of Gbx protein is required for refinement and maintenance of the *Otx2 *expression domain, but not its onset [17,18,55]. Most likely this refinement function in zebrafish and mouse does not act to position the expression domains globally within the neural plate. This level of positioning is controlled by the secreted Wnt8 molecule [32].

The observed sharpening of *otx2 *and *gbx1 *expression domains between 60% and 80% epiboly marks a period during which mutually repressive interactions are likely to take place [8]. Interestingly, a key step in the induction of posterior neural fate in *gbx1*-overexpressing embryos seems to be repression of *otx2 *expression. Our cell transplantation studies have shown that this *otx2 *repression occurs cell-autonomously within the injected cells. Conversely, *otx2 *cell-autonomously represses *gbx1*. These results are consistent with the mutual repression of *Otx2 *and *Gbx2 *observed during mis-expression experiments in mice, *Xenopus *and chicken [17,20,21,25] and suggests that whichever *Gbx *gene is expressed at a higher level or at the proper timing determines cell fate.

### The *otx2/gbx1 *interface in zebrafish is equivalent to the *Otx2/Gbx2 *interface in mouse

Several similarities are observed between zebrafish *gbx1 *and mouse *Gbx2 *(or chicken and *Xenopus*). First, in mouse and chicken, *Gbx2 *is expressed during gastrulation and its expression domain does not immediately abut the *Otx2 *expression domain [55,56]. Only at the end of gastrulation are two distinct expression domains found adjacent to each other. Our previous study [8] has shown that *gbx1 *expression initially overlaps with *otx2 *and then, during gastrulation, refines such that it sharply abuts the *otx2 *domain. Second, in the present work, we show that loss of *gbx1 *does not affect the initial posterior limit of *otx2 *at 60% epiboly, although *gbx1 *is rapidly required to maintain this boundary at 70% epiboly. In mice, it has been shown that the *Otx2 *and *Gbx2 *domains are initially established independently of each other at early headfold stage (E7.75), but that their expression becomes rapidly interdependent by late headfold stage [17,18,55]. Also in chicken, Garda *et al*. [56] described a phase when *Otx2 *and *Gbx2 *overlap in the mid-hindbrain neuroectoderm. This co-expression disappears at HH stage 14–15 and both domains become mutually exclusive and complementary [56]. Third, in mice, misexpression of *Otx2 *and *Gbx2 *during later segmentation stages can shift the MHB organizer [20,57]. We have shown here that raising the dosage of *gbx1 *shifts MHB position, mimicking the mouse *Gbx2 *gain-of-function experiments [57]. Fourth, we also show that ectopic juxtaposition of *otx2 *and *gbx1 *acts to induce MHB markers in the same way as ectopic juxtapositon of *Otx2 *and *Gbx2 *does in the mouse [21,23-25]).

Together with our previous work [8], the present study here illustrates that functional requirements for MHB formation may be achieved differently in zebrafish as compared to other species. Namely, *gbx1*, instead of *gbx2*, may be required early in zebrafish development for the correct specification of the MHB primordium. In zebrafish, it has been shown that *gbx2 *is expressed only at the end of gastrulation, after the MHB is established, in response to Fgf8 signaling [8]. Given that *gbx2 *has the same ability as *gbx1 *to suppress *otx2 *expression in the fore-midbrain region (data not shown) [9], it is possible that positioning of the zebrafish MHB through *otx2 *repression is later reinforced by the expression of a second *gbx *gene. Indeed, the *gbx1 *expression domain begins to fade from the MHB around the five to six somite stage [8]. It is possible that *gbx2 *steps in at this time to maintain the regulatory loops already in place. This is different from the situation in mouse, where only one *Gbx *gene, namely *Gbx2*, is expressed at the MHB and is maintained there during development [30,31].

### *gbx1 *is a posteriorizing factor

Previous work in fish has shown that neural posteriorization is mediated via signals from the marginal blastoderm, the non-axial mesendoderm of pre-gastrula stage embryos [58]. Transplantation of cells from this region into the animal pole of the embryo has previously been shown to induce posterior neural markers, *krox20*, *hoxa1 *or *gbx1*, in the host tissue, suggesting that secreted molecules might be involved in this process [32,58,59]. Wnts, Nodals, retinoic acid and fibroblast growth factors are all good candidates to be posteriorizing molecules (for a review, see [60]). As of yet, experiments addressing this early neural posteriorization have only addressed the function of secreted factors; the role of transcription factors is unexplored. We have recently shown that Wnt8, a known posteriorizing molecule, is required for the initial subdivision of the neuroectoderm, including for the onset of posterior *gbx1 *expression [32]. Here we have shown that *gbx1 *overexpression, like *wnt8 *overexpression, posteriorizes the neuroectoderm in a cell-autonomous fashion and that *gbx1 *overexpression can partially restore hindbrain patterning in the absence of Wnt8. Collectively, our data point to a role for this homeodomain transcription factor in mediating Wnt posteriorizing activity during neuroectoderm development. In this process it likely cooperates with other transcription factors expressed in the margin and which mediate Wnt signaling during gastrulation. Two Sp1-related transcription factors, *sp5 *and *sp5-like*, are known to be direct targets of Wnt signaling and to mediate hindbrain patterning [49]. Furthermore, two caudal-related genes, *cdx1a *and *cdx4*, are expressed in the blastoderm margin during early gastrulation [61] and are regulated by the Wnt/β-catenin pathway [62]. It thus seems likely that the gradient of Wnt activity subdivides the posterior neural plate by activating specific transcription factors at different positions. These subdomains are established at the end of gastrulation (80% epiboly), a stage when hindbrain/spinal cord progenitors are proposed to acquire regional identity [63].

## Materials and methods

### Whole-mount *in situ *hybridization and antibody staining

ISH and antibody detection were performed using protocols described in [39]. Probes and wild-type expression patterns were as previously described for: *krox20 *[64]; *otx2 *[65]; *pax2.1 *[66]; *eng2 *[41,42]; *wnt1 *[40]; *fgf8 *[39]; *gbx1 *and *gbx2 *[8]; *myoD *[67]; *six3 *[68]; *emx1 *[37]; *pax6 *[69]; *hoxa1 *[43]; *cdx4*/*caudal *[45]; *val *[44]; and *hoxb1 *[70]. Antibody staining against acetylated tubulin (Sigma, St Louis, MO, USA)) was carried out as previously described [71].

### Transplantation

Donor embryos were injected with biotin coupled tetramethyl-rhodamine dextran (10,000 MW, Molecular Probes D-1817, Eugene, OR, USA) diluted in 0.25 M KCl. Transplantation of donor cells into host embryos was done at shield stage using trimmed borosilicate capillaries. Transplanted cells were then visualized by immunochemical staining using the Vectastain ABC system (VectorLabs, Burlingame, CA, UK)) and DAB (Sigma).

### Labeling of cell clones via laser-based activation of caged fluorescein

Non-fluorescent, photoactivatable (caged) fluorescein was used as a cell tracer for fate mapping in the zebrafish embryo as previously described [72].

### DNA constructs

cDNAs encoding *gbx1 *or *otx2 *were subcloned into the pCS2+ or pCS2+MT vectors [73].

### RNA and morpholino injection

Capped mRNAs were synthesized as previously described [39]. For RNA injection, embryos were dechorionated using pronase and injected at the one-cell stage. Two antisense morpholino oligonucleotides (Gene-Tools, Inc., Philomath, Oregon, USA) were designed to target *gbx1 *(Mo1 and Mo2): Mo1, 5' AAATCCCGTGCTGTACTGGCCTTCA 3'; Mo2, 5' CGCTGCTGAAGGGTCCTCGCCGTCC 3'. Morpholinos were resuspended in sterile water, stored at -20°C as 10 mg/ml solutions and diluted before use to the appropriate concentration in water containing 0.2% phenol red. Morpholinos were injected in the yolk with a concentration of 1–10 ng between the one- to four-cell stage.

### Western blotting

For western blot analysis, embryos were deyolked and proteins were extracted from injected or non-injected embryos. Protein extracts were resolved by standard SDS-PAGE. Samples were electroblotted onto Protan nitrocellulose (Schleicher and Schuell, Dassel, Germany)). Membranes were incubated in Tris-buffered saline 1% low-fat milk for 1 h at room temperature with the anti-c-myc antibody (9E10) or E7 anti-tubulin ascites (Developmental Studies Hybridoma Bank). Immunocomplexes were revealed by chemiluminescence (Amersham, Buckinghamshire, UK)) with anti-mouse or anti-rabbit immunoglobin G antibodies conjugated to peroxidase (Sigma). Maintenance of and experimentation with zebrafish is covered under permits 24D-9165.40/1-2007 and 24D-9168.11-1/2008-3, and for genetic engineering work under permit 56-8811.71/189 of the State Government of Saxony, Germany, to MB.

## Abbreviations

A-P: antero-posterior; ISH: *in situ *hybridization; MHB: midbrain-hindbrain boundary; Mo: morpholino; rh: rhombomere.

## Authors' contributions

MR conceived the studies, designed and carried out the experiments, analyzed and interpreted the data, and wrote the article. KL performed injection experiments, cell-tracing experiments and analyzed data. RA helped with the injection experiments. MW helped with the ISH. MB obtained the funding, conceived the study, designed the research, analyzed data and wrote the article.

## Supplementary Material

Additional file 1 **Comparison of *gbx1 *Mo1 and Mo2 induced phenotypes**. **(A-F) **Lateral views, anterior to the top (the tailbud stage) and to the left (24 h). **(G-K') **Dorsal views, anterior to the top. (A) Control embryo at the tailbud stage; (B, B') embryos at the tailbud stage injected with 5 ng and 10 ng Mo1 respectively; (C, C') embryos at the tailbud stage injected with 5 ng and 10 ng Mo2 respectively. (D) Control embryo at 24 h; (E) 24 h embryo injected with 5 ng Mo1; (F) 24 h embryo injected with 5 ng Mo2. Click here for file

Additional file 2 ***krox20 *expression at somite stage in *gbx1 *morphants**. **(A) **Control embryo at the seven-somite stage stained with *otx2*/*krox20*/*myoD*. *otx2 *is not seen in this picture. The most anterior expression seen is the *krox20 *domain. *myoD *indicates the number of somites. **(B) **Embryos at the seven-somite stage injected with 7 ng of Mo1. The most anterior expression seen is the border of the *otx2 *domain and *krox20 *is not visible. Click here for file
