# Supplementary figures and images for: Zebrafish gbx1 refines the Midbrain-Hindbrain Boundary border and mediates the Wnt8 posteriorization signal
Source: Neural Dev. 2009 Apr 2;4:12. doi: 10.1186/1749-8104-4-12 (PMC2674439; doi:10.1186/1749-8104-4-12)

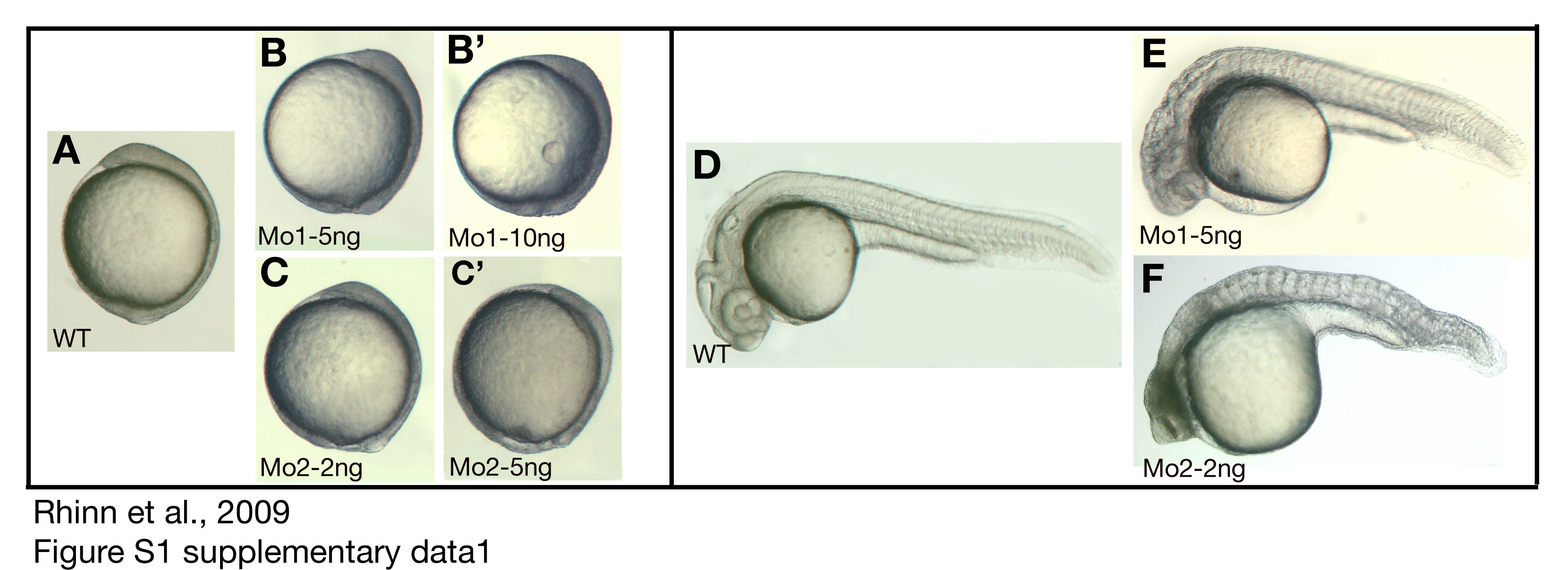

Supplement: Additional file 1 — Comparison of gbx1 Mo1 and Mo2 induced phenotypes. (A-F) Lateral views, anterior to the top (the tailbud stage) and to the left (24 h). (G-K') Dorsal views, anterior to the top. (A) Control embryo at the tailbud stage; (B, B') embryos at the tailbud stage injected with 5 ng and 10 ng Mo1 respectively; (C, C') embryos at the tailbud stage injected with 5 ng and 10 ng Mo2 respectively. (D) Control embryo at 24 h; (E) 24 h embryo injected with 5 ng Mo1; (F) 24 h embryo injected with 5 ng Mo2. [file 1749-8104-4-12-S1.jpeg]

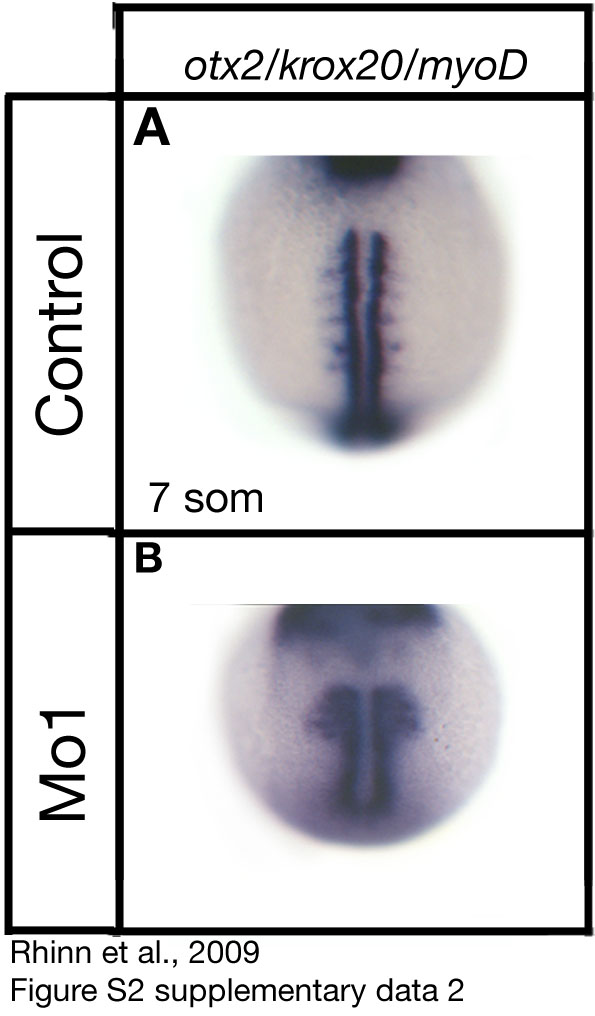

Supplement: Additional file 2 — krox20 expression at somite stage in gbx1 morphants. (A) Control embryo at the seven-somite stage stained with otx2/krox20/myoD. otx2 is not seen in this picture. The most anterior expression seen is the krox20 domain. myoD indicates the number of somites. (B) Embryos at the seven-somite stage injected with 7 ng of Mo1. The most anterior expression seen is the border of the otx2 domain and krox20 is not visible. [file 1749-8104-4-12-S2.jpeg]
